# Uterine Lucorrhæ of Old Women

**Published:** 1861-08

**Authors:** 


*Uterine Leucorrhœa of Old Women.*—This disease is not peculiar to women who have passed the childbearing period of life. Uterine leucorrhœa occurs in young women, in various forms; but in old women, who have for a more or less considerable period ceased to menstruate, it has appeared to Dr. J. M. Dundan to have more characteristic, and, perhaps, peculiar symptoms. Its treatment has also peculiarities; but, above all, its diagnosis is important for two great reasons—first, that it may be appropriately treated; and, second, that the alarm sometimes excited in the patient, and sometimes in the practitioner, by the great similarity of the symptoms to those of cancer, the bane of women of mature years, may be subdued.

Discharge per vaginam of muco-purulent matter is a symptom of the disease. The discharge varies in character, being sometimes like mucus, and thin, sometimes purulent, and more or less viscid. It is occasionally mixed with blood, or only tinged with it. In some cases this sanguinolence is produced only by the heat of the bed, or by anything coming in contact with the cervix uteri, especially if its mucus membrane happens to be abraded. If the discharge is retained in uteri, even for only several hours, it acquires a putrid odor. Its retention is apt to occur from the progressive atrophy of the neck of the womb leading to contraction of its canal. It may also, in some cases, be the result of flexion of the uterus; the influence of gravitation being then occasionally superadded to the dimensional contraction of the cervical canal at the seat of the flexion.

When the discharge does not flow freely, but accumulates in and distends the cavity of the uterus, it gives rise to a peculiar pain around the loins or pelvis, of a girding nature, as if a tight, hard cord partially or entirely encircled the person—a pain having probably some remote analogy to the corresponding symptom produced when labor is obstructed by the distended hydrocephalic head pressing on the cervix uteri. Other pains may be present in the region of the uterus, or there may be irritation and pain of the vulva, from the constantly passing discharge; but these are not characteristic symptoms.

The only other notable symptom is disorder of the stomach and vomiting. When it occurs, it is evidently the result of what is called sympathy with, or the reflected action of, the uterine nerves irritated by a replete and tense uterine cavity.

The exact seat and nature of the disease requires for its diagnosis a careful physical examination. The more or less atrophied and tent-shaped fornix of the vagina is first felt, and at its apex the more or less atrophied cervix, with a patulous mouth. The body of the uterus generally stands high, and may be felt to be enlarged, generally, though not always, inconsiderably. A probe, passed into the patulous external os, soon finds that the internal os uteri is not in a similar condition. But having permeated it, the uterine cavity is found to be wide and capacious, the point of the probe moving preternaturally freely in it. When the probe passes without foice, it causes almost no pain. It should be urged and handled with great care and gentleness; for, should the uterine walls have their toughness and elasticity destroyed by disease, whether simple or malignant, a probe may easily accidentally wound, or even transpierce them; and, while such a wound may be harmless in the case of healthy walls, its gaping condition, when made in an unelastic wall, will render it at least dangerous, and probably fatal.

If a small plug of sponge-tent be passed into the cervical canal, to dilate it for the free passage of the discharge, the latter will be restrained completely for the time, and the girding pain will be much increased. On the removal of the sponge, the discharge will come away fetid in a gush, and the girding pain will be completely relieved. Much care is necessary in using the sponge; for, if too large, it may lacerate the rigid atrophied cervix by its rapid expansion; or, by too long obstruction of the discharge, the over distended uterus may burst, especially if its walls are degenerated and unelastic; or, by the same cause, the noxious fluid may be forced through the tubes into the peritoneal cavity. By means of a speculum an abraded condition of the cervix uteri may be remarked; and probably the process of the examination will cause blood to ooze from these parts.

The general aspect of cases of uterine leucorrhœa in old women appears to differ considerably from that of the young, although there is no single feature to distinguish them if the atrophy of age be omitted. The last condition implies a smooth vagina contracting in dimensions in its upper part, an elevated uterus, a small cervix—states which are, of course, never observed in the young. But it will be found that in the old the disease is more chronic than in the young; that there is less pain and tenderness in the old than in the young; that in the old, thickened uterine walls and flexions or versions are rarer than in the young; that sanguinolence of the discharge is also rarer in the old than in the young; while fetor of it is more common, from a circumstance already mentioned, which leads to its more prolonged retention in utero. All these differences may not exist in any two cases that may come under a practitioner's care; and even if they did, they would not be sufficient to establish any essential difference in the diseases; but they are of considerable importance nevertheless.

The treatment which has proved most successful in the hands of the author, is one which is certainly not generally applicable to cases occurring in young women. It is the regular use of cauterization by nitrate of silver, applied every third or fourth day to the interior of the uterus, in Lallemand's porte-caustic. After each application the discharge is altered in character for a day, and subsequently diminished in quantity till it gradually disappears. Another remedy appeared to be of marked service, namely, irrigation of the cervix uteri and vagina with water considerably below the temperature of the body. This is easily effected by a Higginson's syringe, a syphon, or some other suitable apparatus.

As the cure progresses, the dimensions of the cavity of the uterus are perceived, on introduction of the porte-caustic, to be gradually lessening, the atrophy of the cervix rapidly increases, the external os uteri loses its patency, and at last the discharge entirely ceases to flow.—*Edinburgh Med. Jour., March*, 1860.

